# Citizen science in the marine environment: estimating common dolphin densities in the north-east Atlantic

**DOI:** 10.7717/peerj.8335

**Published:** 2020-02-28

**Authors:** James R. Robbins, Lucy Babey, Clare B. Embling

**Affiliations:** 1 ORCA, Portsmouth, UK; 2 School of Biological and Marine Sciences, Plymouth University, Plymouth, UK

**Keywords:** Citizen science, Cetacean, Platforms of opportunity, Common dolphin, Bycatch, Distance sampling, Density surface model

## Abstract

**Background:**

Citizen science is increasingly popular and has the potential to collect extensive datasets at lower costs than traditional surveys conducted by professional scientists. Ferries have been used to collect data on cetacean populations for decades, providing long-term time series for monitoring of cetacean populations. One cetacean species of concern is the common dolphin, which has been found stranded around the north-east Atlantic in recent years, with high numbers on French coasts being attributed to fisheries bycatch. We estimate common dolphin densities in the north-east Atlantic and investigate the ability of citizen science data to identify changes in marine mammal densities and areas of importance.

**Materials and Methods:**

Data were collected by citizen scientists on ferries between April and October in 2006–2017. Common dolphin sightings data from two ferry routes across three regions, Bay of Biscay (*n* = 569); south-west United Kingdom to the Isles of Scilly in the Celtic Sea (*n* = 260); and English Channel (*n* = 75), were used to estimate density across ferry routes. Two-stage Density Surface Models accounted for imperfect detection, and tested the influence of environmental (chlorophyll *a*, sea surface temperature, depth, and slope), spatial (latitude and longitude) and temporal terms (year and Julian day) on occurrence.

**Results:**

Overall detection probability was highest in the areas sampled within the English Channel (0.384) and Bay of Biscay (0.348), and lowest on the Scilly’s route (0.158). Common dolphins were estimated to occur in higher densities on the Scilly’s route (0.400 per km^2^) and the Bay of Biscay (0.319 per km^2^), with low densities in the English Channel (0.025 per km^2^). Densities on the Scilly’s route appear to have been relatively stable since 2006 with a slight decrease in 2017. Densities peaked in the Bay of Biscay in 2013 with lower numbers since. Densities in the English Channel appear to have increased over time since 2009.

**Discussion:**

This study highlights the effectiveness of citizen science data to investigate the distribution and density of cetaceans. The densities and temporal changes shown by this study are representative of those from wider-ranging robust estimates. We highlight the ability of citizen science to collect data over extensive periods of time which complements dedicated, designed surveys. Such long-term data are important to identify changes within a population; however, citizen science data may, in some situations, present challenges. We provide recommendations to ensure high-quality data which can be used to inform management and conservation of cetacean populations.

## Introduction

Citizen science has been growing in popularity in recent years, and projects often have hundreds, or thousands of active volunteers collecting data across wide geographical areas and long time periods (Hyder et al., 2015). Long-term monitoring such as this can provide an early warning system of change in the marine environment. Citizen science has been used to study a variety of taxa, for example, birds (Sullivan et al., 2009), intertidal organisms (Vermeiren et al., 2016), or record a broad range of animals across taxa and ecosystems (Postles & Bartlett, 2018). Several citizen science projects collect data on marine mammals, with many of these using shore-based data collection methodologies (Tonachella et al., 2012; Embling, Walters & Dolman, 2015). Vessel-based methods are often restricted to ad hoc data collection of animal presence; however, some studies have successfully used platforms of opportunity (vessels that undertake non-scientific voyages along predetermined routes such as ferries or cruise ships) to undertake citizen science surveys at sea (Williams, Hedley & Hammond, 2006; Kiszka et al., 2007). The use of such platforms is considerably cheaper than chartering a ship and paying running costs, although surveyors have limited or no control over the journey that the vessel undertakes. Such surveys can be used to investigate animal distribution and relative abundance.

An understanding of animal occurrence and areas of importance is critical for potential anthropogenic impacts to be understood, for appropriate conservation management. Standardized and appropriate methods can allow for citizen science data to be used in abundance estimates (Davies et al., 2013), which is key for monitoring species trends in space and time. However, even with standardized methods, it is often challenging for citizen science data to be reliable and accurate enough (Crall et al., 2011) to provide good estimates of abundance due to the difficulties of detecting animals, especially at sea (Buckland et al., 2001). For example, marine mammals spend only a fraction of their time at the surface of the water where they are available to be recorded by vessel-based surveyors (Mate et al., 1995). Animals are also less likely to be recorded at increasing distances from the observer, with probability likely to decrease in worsening conditions (Buckland et al., 2001, 2015), such as higher sea states and swell, reduced visibility, or less experienced surveyors. These uncertain detection probabilities can be estimated and accounted for with distance sampling analysis (Buckland et al., 2001).

The citizen science charity, ORCA, has been using platforms of opportunity to collect data on cetacean occurrence since 1995, with considerable survey effort being undertaken on-board ferries around the UK and north-eastern Atlantic. Data are collected following line-transect distance sampling techniques, which can be used in designed and opportunistic surveys to estimate the abundance and distribution of cetaceans. Designed surveys follow randomly-placed systematic transects to provide a representative coverage of the survey area (Thomas et al., 2010). These surveys can be expensive and time-consuming as they use dedicated ships or aircraft to survey large areas (Hammond et al., 2001, 2013, 2017). As a result, they are often carried out infrequently and provide a snapshot of abundance over a short temporal scale. For example, Small Cetaceans in European Atlantic waters and the North Sea (SCANS) surveys are conducted every 10 years but cover expansive areas and use robust methodologies (Hammond et al., 2001, 2013, 2017). Alternatively, distance sampling surveys can be undertaken with non-random coverage from platforms of opportunity. Due to the non-random nature of these transects, results cannot be extrapolated beyond the surveyed area unless the transects are representative (characterized by being unbiased) of the wider area, unlike designed surveys which use sampling designs that are likely representative. Platforms of opportunity often operate year-round however, and data can be collected on much finer temporal-scales, usually at reduced cost.

This study focuses on short-beaked common dolphins (*Delphinus delphis*, *Linnaeus*; hereafter referred to as common dolphins), in the English Channel, Bay of Biscay, and a small section of the Celtic Sea between Penzance, south-western United Kingdom and the Isles of Scilly. Previous studies suggest that common dolphins are most abundant in the Bay of Biscay, with fewer recorded in the Celtic Sea and English Channel (MacLeod, Brereton & Martin, 2009; Hammond et al., 2017). There is concern about common dolphins in these waters due to an increasing number stranding on European Atlantic beaches in recent years, with many likely to be a result of fisheries bycatch in the Bay of Biscay and Celtic Sea (Crosby et al., 2016; Peltier et al., 2016, 2017). Common dolphins are one of the most frequently bycaught species in north-east Atlantic fisheries (De Boer et al., 2008; Peltier et al., 2016), historically reported in pelagic fisheries targeting sea bass or albacore tuna in the English Channel and Bay of Biscay (Rogan & Mackey, 2007; Spitz et al., 2013). Analysis derived from stranding records and accounting for drift dynamics estimated between 2,250 and 5,750 animals are bycaught per year in the Bay of Biscay, western English Channel and Celtic Sea (Peltier et al., 2016). Given the infrequency of designed surveys in these areas of numerous strandings and high bycatch mortality, citizen science is an ideal method to collect longer term data on the distribution and densities of common dolphins in this high-risk area.

This study uses citizen science data to estimate common dolphin densities in the English Channel, Bay of Biscay and a small part of the Celtic Sea between Penzance (south-west United Kingdom) and the Isles of Scilly, accounting for imperfect detection. Results derived from these citizen science data are compared to published results from robust designed distance sampling surveys undertaken by professional scientists. Temporal variation in common dolphin densities is discussed in relation to mass mortality events and bycatch within the study area. The strengths and limitations of citizen science data are discussed, and recommendations given for accurate and robust citizen science monitoring data.

## Materials and Methods

### Survey area

**Figure 1:**
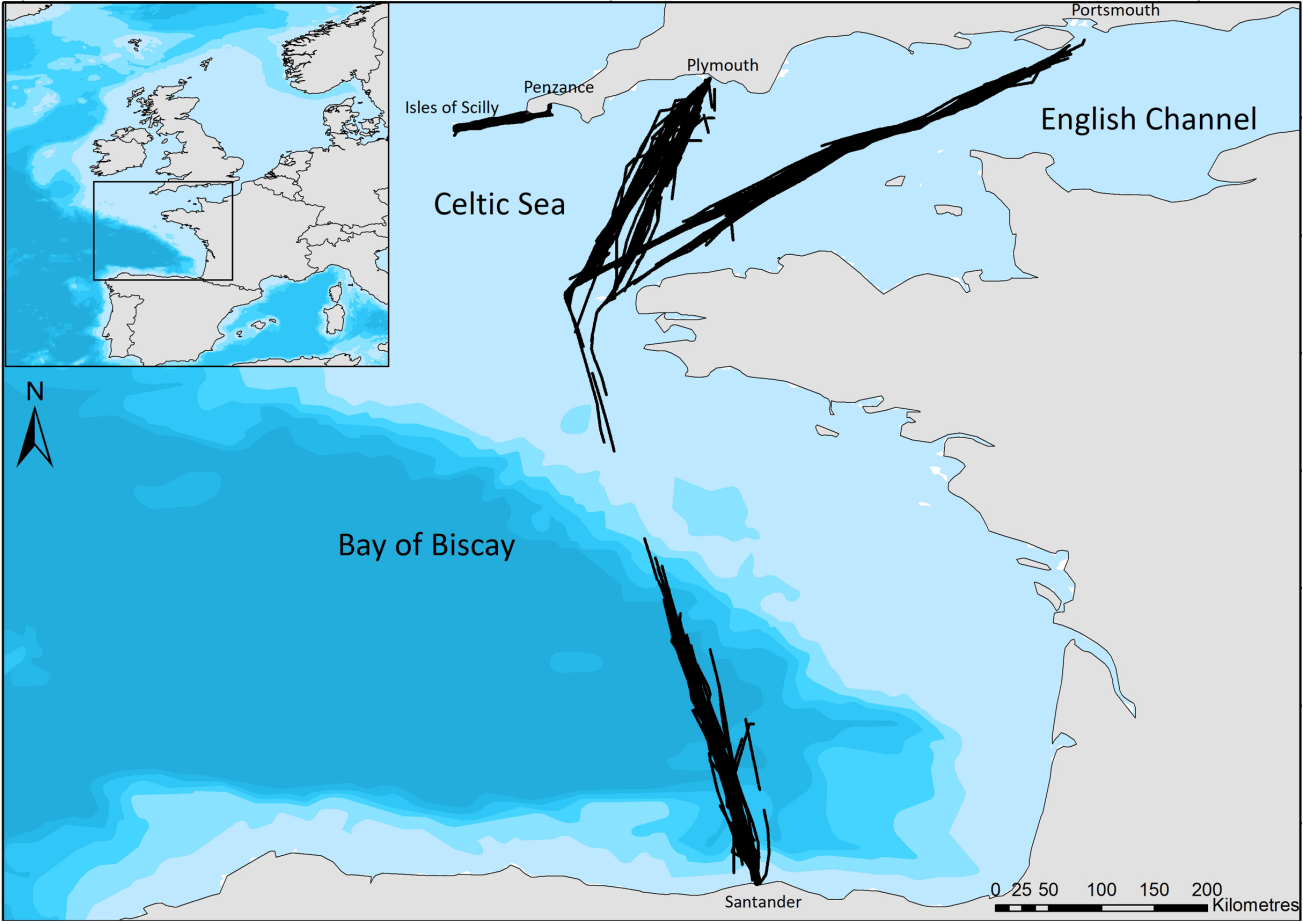
The ferry routes travelled between Plymouth—Santander—Portsmouth through the English Channel and Bay of Biscay, and from Penzance—St Mary’s in the Celtic Sea. Black lines indicate the line of ferry travel when surveyors are actively searching for dolphins. Bathymetry is indicated with light blue to dark blue in order of increasing depth (Bathymetry vector courtesy of Natural Earth: www.naturalearthdata.com).

Our study regions include the Bay of Biscay, a heterogeneous area incorporating relatively shallow coastal areas, the continental shelf edge, and deep-water canyons (Certain et al., 2008); a relatively small, shallow area of the Celtic Sea between Cornwall and the Isles of Scilly, hereafter referred to as the Scilly’s route; and the English Channel, a busy shipping region (McClellan et al., 2014), with relatively shallow waters. Surveys cover ferry routes of Brittany Ferries’ Pont-Aven (21.6 m bridge height) which leaves Plymouth, travels across the English Channel and the Bay of Biscay to Santander, and then returns to Portsmouth (Fig. 1). No survey effort was undertaken on the southern edge of the continental shelf due to the ferry crossing this area at night. The Isles of Scilly Travel’s Scillonian III (10 m bridge height) crosses from Penzance to St Mary’s on the Scilly Isles in the Celtic Sea (Fig. 1). Regions of the English Channel (inter-region boundaries east of 5°W and north of 48°N), Bay of Biscay (south of 48°N) and Scilly’s route (west of 5°W) are referred to henceforth for ease; however, the area sampled is constrained to ferry routes that cross these and may not be representative of the entire area.

### Data collection

Data were collected by trained citizen scientists between 2006 and 2017, with survey effort concentrated between April and October, and no surveys conducted between November and February. Only data collected during April–October were used in the analysis due to a similar number of surveys throughout this period. Frequency of surveys varied across the study period but averaged once per month on the Plymouth—Santander—Portsmouth route and twice a month on the Penzance—Isles of Scilly route. Trained surveyors were deployed on ferries by ORCA (www.orcaweb.org.uk) and collected data from the forward-facing bridge of vessels according to standard distance sampling methodologies (Buckland et al., 2001, 2015). Survey teams comprised of four surveyors on the Pont-Aven (at least three of which were experienced), allowing for 30-minute rest breaks to avoid observer fatigue, and three on the Scillonian III (at least two of which were experienced) due to shorter survey lengths. Two observers scanned the forward 180° (100° each, with a 10° crossover at the trackline). The data recorder collected effort data, including environmental conditions (glare, sea state, swell, precipitation, and visibility) at a minimum of 30-minute intervals, or when conditions changed, and sighting event details on cetacean species. Group sizes were estimated, and angles from the ships’ bow to animals were recorded using an angle board. Radial distances were calculated from reticle binoculars where possible, or alternatively estimated by eye.

### Data analysis and detection function modelling

Observer eye height (height of reticle above the sea), was determined to be the height of the platform in addition to the height of the average United Kingdom adult (1.68 m). Distances calculated from reticle readings were used to calculate perpendicular distance where available; however, distances estimated by eye were also included only if closer than 250 m, due to distance estimation being difficult at sea, especially at greater distances (Gordon, 2001). Perpendicular distances from the trackline were over-inflated at 0 m (i.e., on the trackline) due to a prevalence of angles being rounded to 0°. As a result, exact perpendicular distances were converted into “bins,” for example, all sightings between 0 and 268 m are in the first “bin,” with “cutpoints” at 0 and 268 m.

A two-stage Density Surface Modelling (DSM) approach was adopted, first by fitting a detection function to obtain detection probabilities for common dolphins. The results of this function were used in a Generalized Additive Model (GAM), with the per-effort segment count of individuals as the response to calculate estimated number of animals, and the relationship with tested covariates. This approach is well documented in Miller et al. (2013) and Buckland et al. (2015). Detection functions estimate the probability of detecting animals a given distance from the line, or *g*(*y*), where *y* is the perpendicular distance, and allow the influence of covariates such as environmental conditions on detection probability to be tested. Distance sampling analysis was carried out in R (R Development Core Team, 2017). It was assumed that animals were detected at their initial location, prior to responsive movement; however the survey design did not allow this to be tested. It was assumed that common dolphins on the trackline were always observed, *g*(0) = 1, or close enough to have little impact on the results, based on quick dive times, and often clear surface behaviors (Hammond et al., 2001; Cañadas & Hammond, 2008; Becker et al., 2010). The final assumption of line-transect distance sampling is that measurements are taken accurately, which was violated by rounded angles.

**Figure 2:**
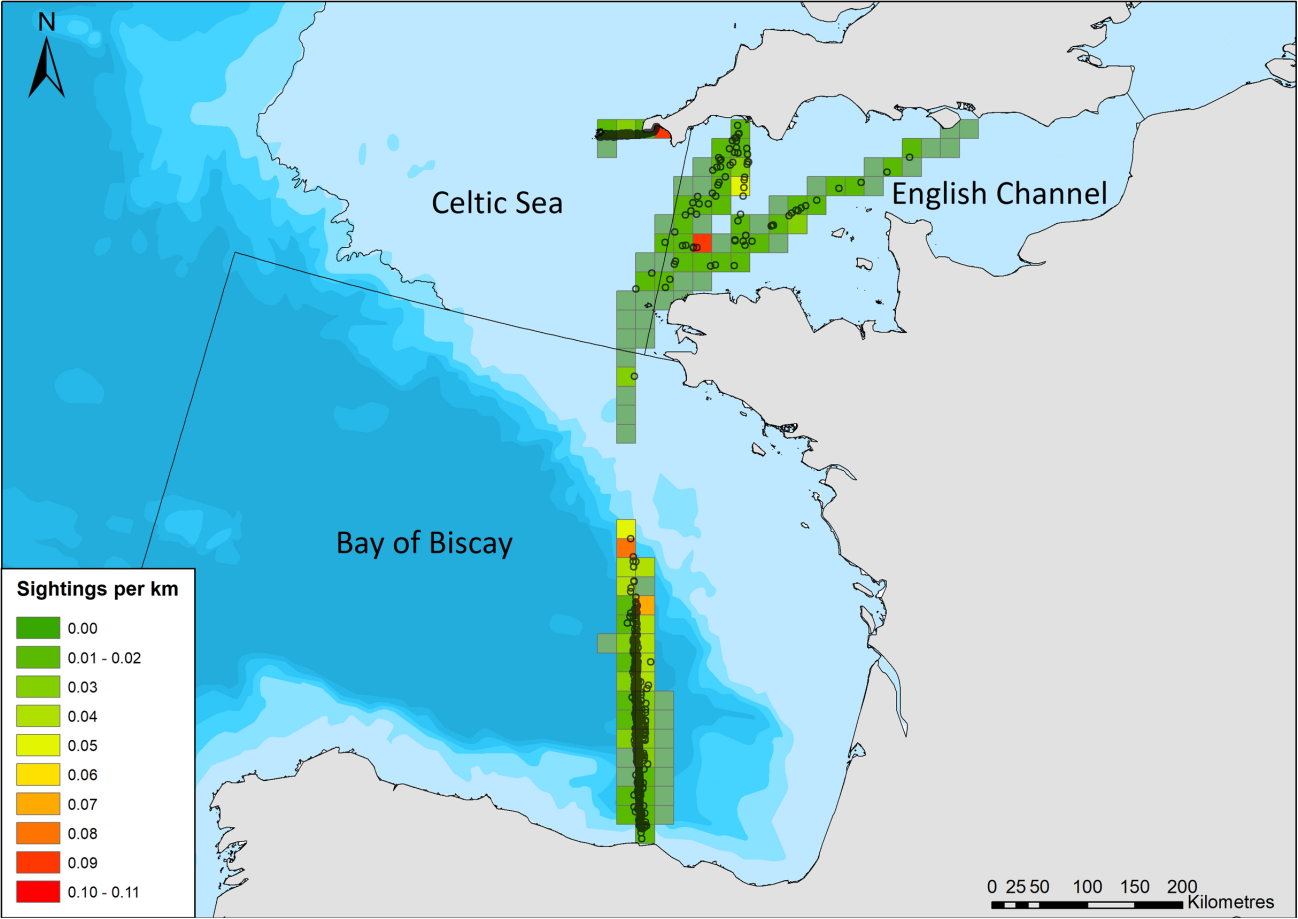
Common dolphin sightings across the study area. Black lines crossing water depict region boundaries for the English Channel, Celtic Sea, and Bay of Biscay. Open circles show locations of common dolphin groups. Grid cell color represents common dolphin groups per km of effort. Bathymetry is shown, with sightings in shallow water (light blue), through to waters up to 4,000 m deep (dark blue). Bathymetry vector courtesy of Natural Earth: www.naturalearthdata.com.

Detection functions were originally fitted (distance package; Miller, 2017) for a single dataset with all routes and years combined; however, region was found to alter detectability, likely due to varying platform heights. As a result, regions (as defined by sea regions: English Channel, Celtic Sea (Scilly’s route), and Bay of Biscay and Iberian Coast; Fig. 2) were stratified, and detection functions and density surface models were fitted for each region separately. A range of detection function models were fitted including hazard rate, and half normal forms, and including up to three covariates that may influence detection probability: group size; region (when the entire dataset was modelled as a whole); sea state; precipitation; visibility; vessel speed; and platform height. The effect of truncation distances and cut points on the detection functions was also investigated. Subsets of detection functions were selected that were deemed to have an adequate fit, based on chi squared goodness of fit tests. The best model for each region was selected based on minimizing the Akaike Information Criterion (AIC) score, and was carried forward to account for imperfect detection in DSM estimates.

### Density estimation

Transects were segmented into approximately 5 km lengths using Marine Geospatial Ecology Tools (Roberts et al., 2010) for ArcMap 10.5 (ESRI, 2017). Environmental covariates which have influenced common dolphin occurrence in previous studies (Table 1): latitude, longitude (Cañadas et al., 2005), depth (Cañadas & Hammond, 2008), sea surface temperature (SST; Moura, Sillero & Rodrigues, 2012), distance to coast (Cañadas & Hammond, 2008), slope (Cañadas, Sagarminaga & García-Tiscar, 2002), and chlorophyll concentration (*chl*_*a*_; Moura, Sillero & Rodrigues, 2012) were assigned by segment centroids with ncdf4 and raster packages (Pierce, 2017; Hijmans, 2017, respectively). The number of individuals, corrected for imperfect detection, was estimated for each segment. GAMs (Wood, 2006) allow for non-normal response data, such as count/abundance of a species, to be related to the predictor variables using non-parametric smooths and were used to model relative abundance (referred to as “abundance” henceforth) with DSMs, whilst accounting for imperfect detection (Miller et al., 2017). Segments were used as a grid in spatial models, with each cell length equal to approximately 5 km (measured in Europe Albers equal conic area projection), and width equal to the truncation distance of the appropriate regions’ detection function.

**Table 1:** Summary of the key environmental covariates used in the DSM, their source and resolution. Sea surface temperature and chlorophyll data are monthly composites for the appropriate year.

Covariate	Source	Approximate resolution
Depth at mean tide height	EMODnet Bathymetry Consortium (2016)	463 m^2^
Sea surface temperature	MODIS Aqua level 3; NASA, Ocean Biology Ecology Laboratory (2017)	4 km^2^
Chlorophyll	MODIS Aqua level 3, OCI algorithm; NASA, Ocean Biology Ecology Laboratory (2017)	4 km^2^
Distance to coast	Calculated with Albers equal European projection in ArcMap (ESRI, 2017)	
Slope	Calculated from min & max depth values (EMODnet Bathymetry Consortium, 2016)	463 m^2^

One-way thin plate regression smooths and two-way tensor smooths were used to model abundance with the spatial covariates, with a one-way smooth of environmental covariates, using the mgcv package (Wood, 2006). Models were compared between those based on a negative binomial distribution and a Tweedie distribution which adequately handles zero-inflated spatial models (Miller et al., 2013). The number of allowed knots (*k*) in the smooth was varied up to *k* = 15 to investigate the best model fit, whilst EDF were considered in order to avoid overfitting models. The best model was selected based on minimising the AIC score, including only those variables that were significant to *p* < 0.05 according to step-wise model selection. Plots of randomized quantile residuals, and residuals against fitted values were checked for normality, auto-correlation and homoscedasticity. Abundance was estimated with a Horvitz–Thompson-like estimator which accounts for detection probabilities arising from count data (Miller et al., 2018). Density was calculated by estimated abundances divided by segment cell area and is reported as number of individuals per km^2^. For each estimate, the coefficient of variance (CV) and 95% confidence intervals (95% CI) were calculated by variance propagation, including uncertainty arising from the detection function, and GAMs (Miller et al., 2013). Density estimates for the Scilly’s route were compared to those from models that only included the outward leg from Penzance to St Mary’s, but not the return, to investigate whether returning across the same area in quick succession influenced results, and to check model performance.

## Results

There were 969 sightings of 11,993 common dolphins during the 68,206 km of effort undertaken by citizen scientists between March and October 2006–2017. The amount of effort and sightings fluctuated considerably between years, with a generally increasing trend in the amount of effort over time (Table 2). The majority of sightings were in the Bay of Biscay (Fig. 2), with 611 sightings, of 8,287 animals (group size range = 1–1,000, median = 8). There were 273 sightings of 2,516 animals on the Scilly’s route (group size range =1–150, median = 6), and 85 sightings of 1,190 animals in the English Channel (group size range = 1–200, median = 6).

**Table 2:** Number of sightings and effort to the nearest km for each survey region and year.

Survey region	Data	2006	2007	2008	2009	2010	2011	2012	2013	2014	2015	2016	2017	Total
English Channel	Effort	640	1183	1529	2507	2404	2661	2390	2091	2726	2261	1705	2167	24262
	Sightings	1	2	5	0	1	4	6	9	11	3	9	34	85
	Sightings per km	0.0015	0.0016	0.0032	0	0.0004	0.0015	0.0025	0.0043	0.0040	0.0013	0.0052	0.0156	0.0035
Scilly’s route	Effort	274	196	138	783	2005	1603	1507	1671	1959	2011	1768	1997	15915
	Sightings	0	1	3	5	4	18	14	49	14	74	79	12	273
	Sightings per km	0	0.0051	0.0217	0.0063	0.0019	0.0112	0.0092	0.0293	0.0071	0.0367	0.0446	0.0060	0.0171
Bay of Biscay	Effort	1200	2173	2808	2766	2276	2797	2618	2212	2424	2391	1721	2643	28029
	Sightings	25	39	40	2	6	49	65	75	78	110	53	69	611
	Sightings per km	0.0208	0.0179	0.0142	0.0007	0.0026	0.0175	0.0248	0.0339	0.0321	0.0460	0.0307	0.0261	0.0217

### Probability of detection and density estimates

#### English Channel

**Figure 3:**
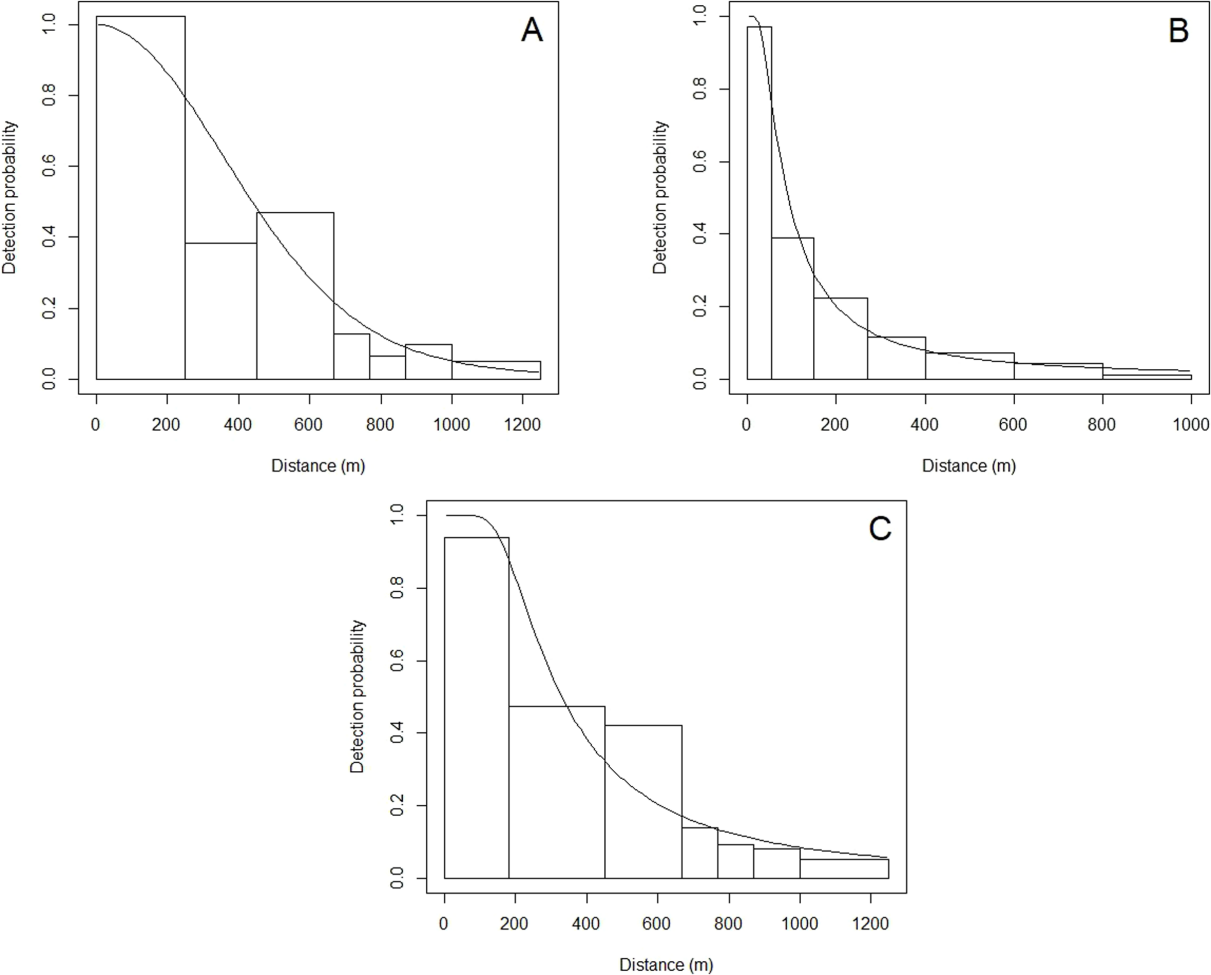
Detection functions showing the detection probability of common dolphins at perpendicular distances (m). (A) English Channel, (B) Scilly’s route, (C) Bay of Biscay.

A total of 24,262 km of effort was undertaken in the English Channel, with at least 2,000 km in most years, with reduced effort (less than 2,000 km) in 2006–2008, and 2016 (Table 2). The best detection function was a half-normal, including 75 sightings within the truncation distance of 1,250 m (Table 3). Vessel speed, sea state, and group size were retained in the model as they affected detection probabilities, with higher vessel speeds, higher sea state, and lower group sizes resulting in reduced probability of detection. This resulted in an average probability of detection of 0.384 within the truncation distance (Fig. 3). The best density surface model was Tweedie, and included a 2-way smooth of longitude and latitude (*p* = 0.04) and year (*p* = 0.03), explaining a relatively low 13.2% of deviance but passed model checks for fit, normality, auto-correlation and homoscedasticity. Density was estimated to be 0.025 common dolphins per km^2^ (95% CI [0.016–0.040]), with a coefficient of variation (CV) of 0.229 (Fig. S1).

**Table 3:** Final detection function models for the English Channel, Scilly’s route, and Bay of Biscay.

Region	Model	Truncation distance (# sightings)	*p* (SE)	ESW (SE)	% CV	Variables
English Channel	Half-normal	1250 m (75)	0.384 (0.04)	480 (50.62)	10.6	Vessel speed + sea state + group size
Scilly’s route	Hazard-rate	1000 m (260)	0.158 (0.019)	158 (19)	12.2	Sea state + group size
Bay of Biscay	Hazard-rate	1250 m (569)	0.348 (0.02)	435 (2.5)	5.9	Vessel speed, sea state + group size

**Figure 4:**
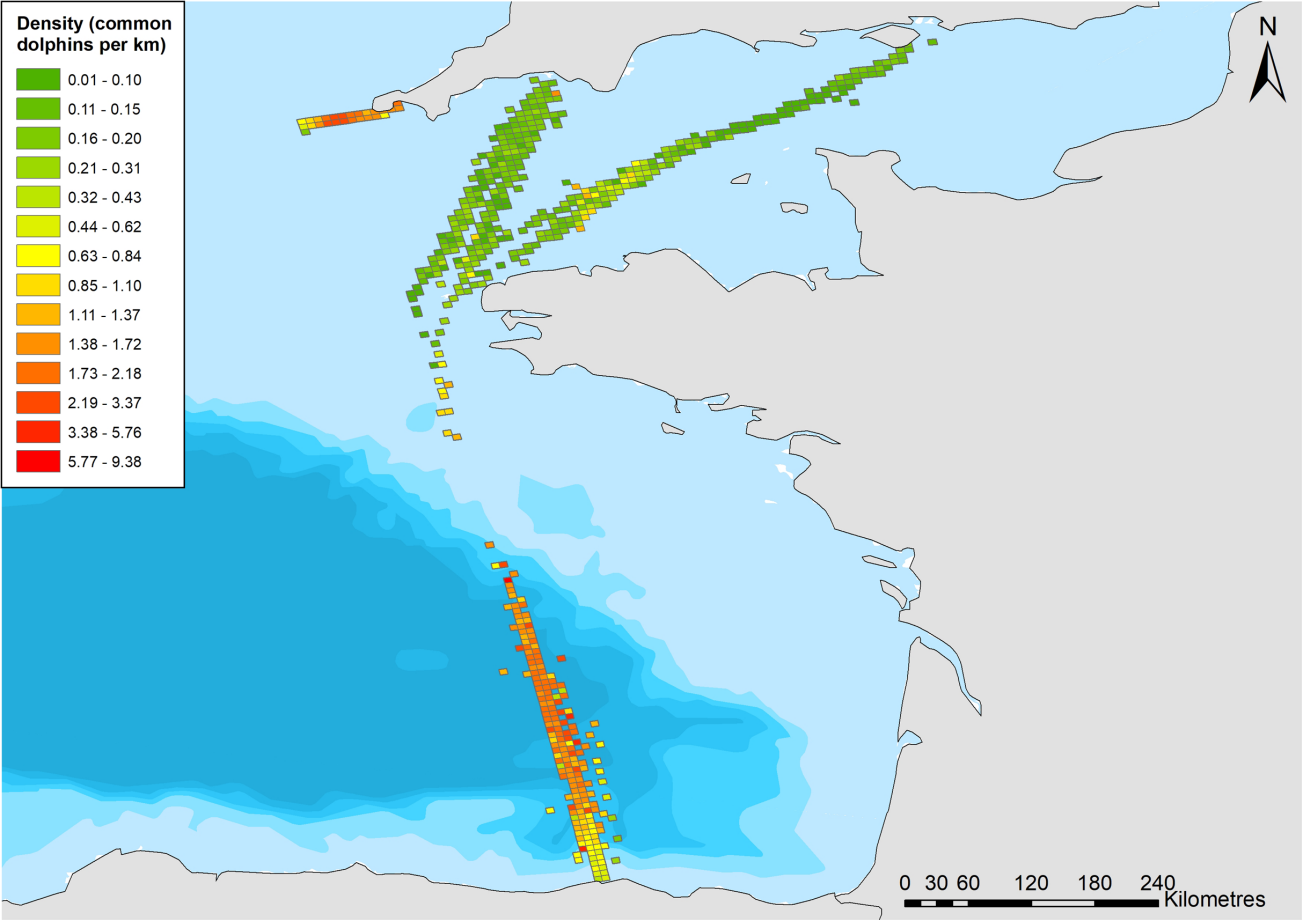
Density of common dolphins (per km ^2^) across the study area. Bathymetry is indicated with light blue to dark blue in order of increasing depth (Bathymetry vector courtesy of Natural Earth: www.naturalearthdata.com).

**Figure 5:**
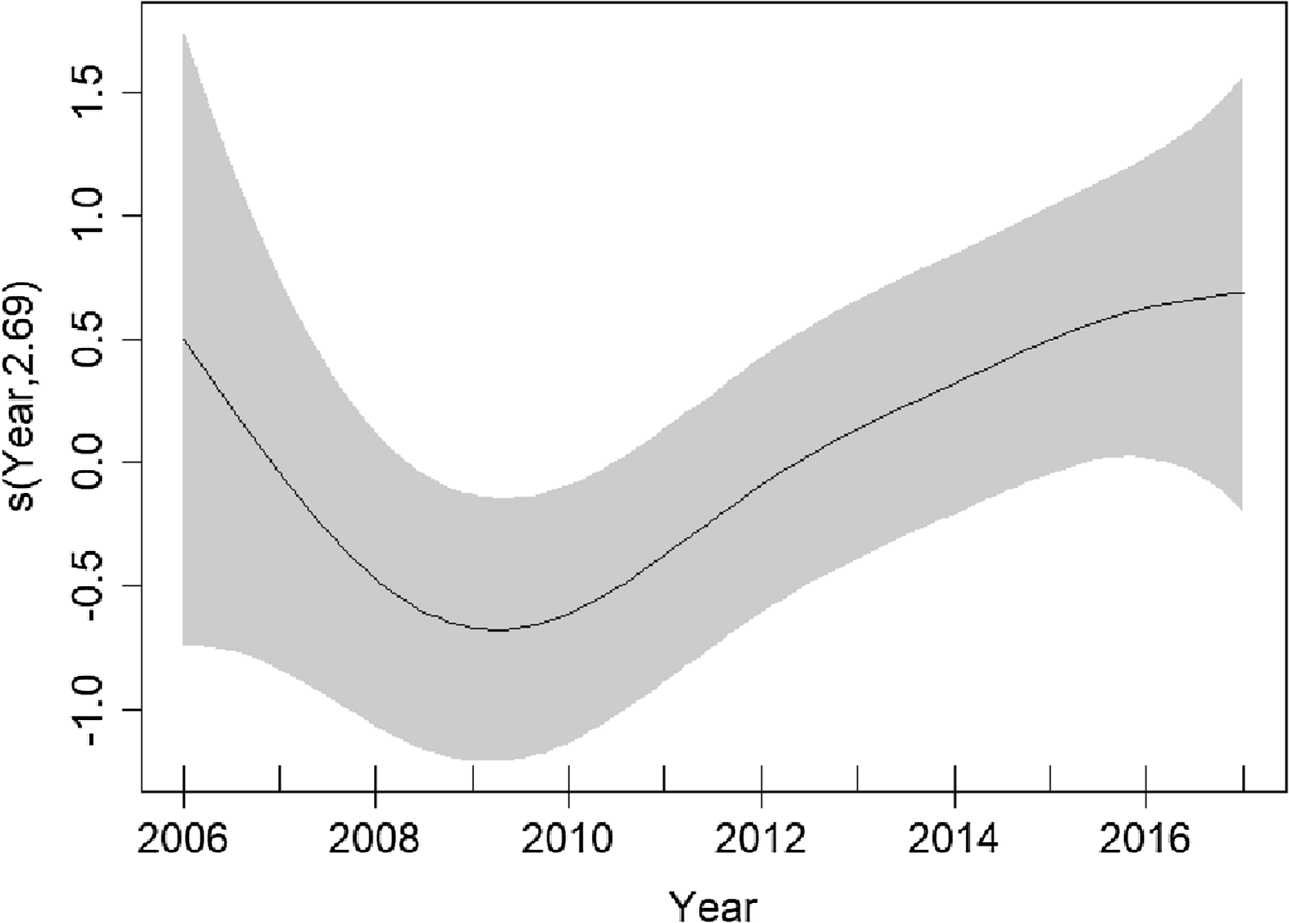
Plots of the GAM smooth fit of abundance between years in the English Channel. Solid line represents the best fit, with the gray shaded area representing the 95% confidence intervals. Vertical lines on the *x*-axis are the observed data values.

Higher densities were predicted to occur ~20 km north of the Finistere region of Brittany (Fig. 4). Variation between years is uncertain due to wide confidence intervals; however, it appears that densities decreased from 2006 to 2009 and have been increasing since (Fig. 5).

#### Scilly’s route

A total of 15,915 km was travelled whilst searching for cetaceans along the Scilly’s route, with reduced effort in 2006–2009 (Table 2). The best hazard-rate detection function included 260 sightings within the truncation distance of 1,000 m (Table 3). Group size and sea state were retained, with larger group sizes, and lower sea states resulting in improved detection probabilities. The average detection probability was relatively low compared to other regions at 0.158 (Table 2).

**Figure 6:**
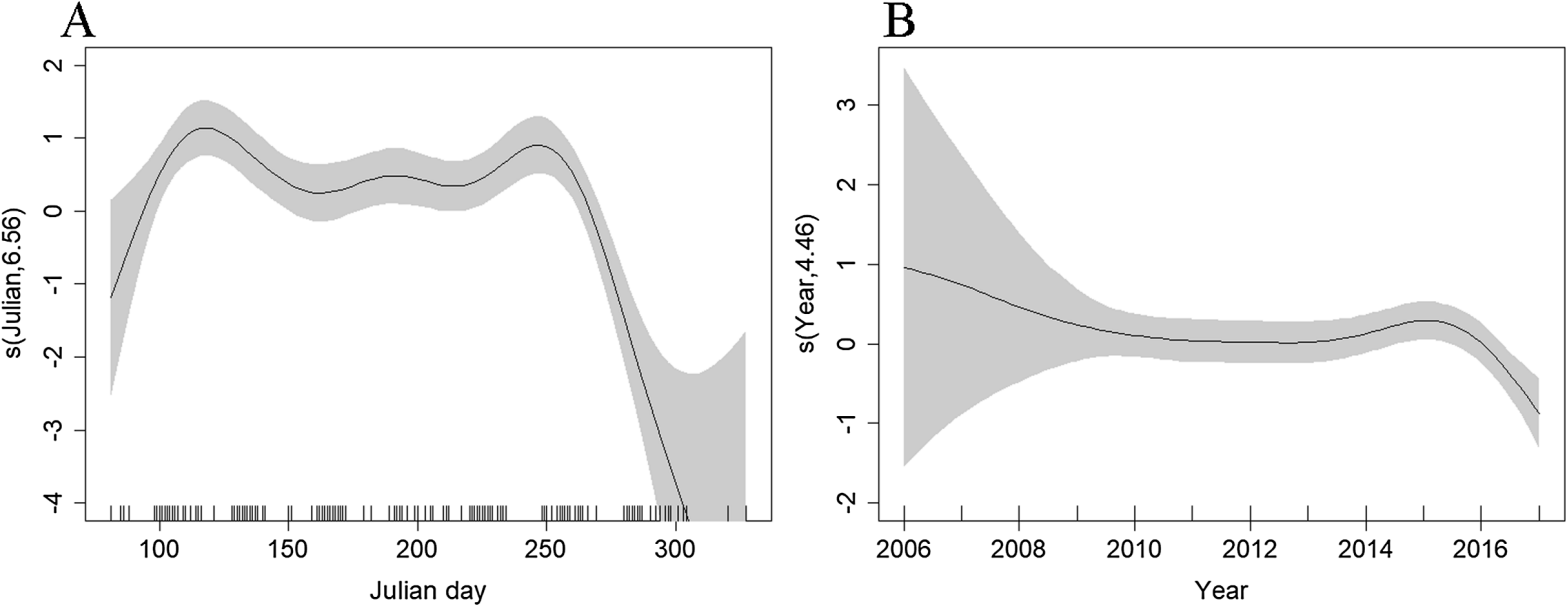
Plot of the GAM smooth fit of abundance between (A) Julian days, and (B) Years on the Scilly’s route. The solid line represents the best fit, with the gray shaded area representing the 95% confidence intervals which are wide between 2006–2008 and early spring and late autumn when effort is low. Vertical lines on the *x*-axis are the observed data values.

The best density surface model was Tweedie and included a 2-way smooth of latitude and longitude (*p* < 0.001), and 1-way smooths of chlorophyll (*p* < 0.001), year (*p* = 0.004) and Julian day (*p* < 0.001) explaining 23.2% of deviance. There was an estimated density of 0.400 common dolphins per km^2^ (95% CI [0.305–0.524]), with a coefficient of variation of 0.139 (Fig. S2). The highest densities were predicted to occur in the middle of the route, ~20 km east of the Isles of Scilly (Fig. 4). Densities have been fairly stable over time, with a decrease in 2017 (Fig. 6B). Densities decreased towards winter, with stable numbers throughout summer (Fig. 6B). The influence of chlorophyll concentrations was significant, with a slight decrease in density associated with higher concentrations, however confidence intervals are wide, resulting in a high degree of uncertainty (Fig. S3). Densities were similar between models that included both the outward and return journey (0.40 dolphins per km^2^), and models that only included a single leg (0.39 per km^2^), suggesting suitable performance and limited influence of repeated journeys within quick succession.

#### Bay of Biscay

A total of 28,029 km of effort was undertaken in the Bay of Biscay from 2006 to 2017, with reduced effort in 2006 and 2016 (Table 2). The best model was a hazard-rate key function, with 569 sightings included within the truncation distance of 1,250 m (Table 3). Speed, sea state, and group size were retained in the detection function as they affected detection probability, with higher speeds, higher sea states, and smaller group sizes reducing detection probabilities. The average probability of detection was 0.348 (Fig. 2).

**Figure 7:**
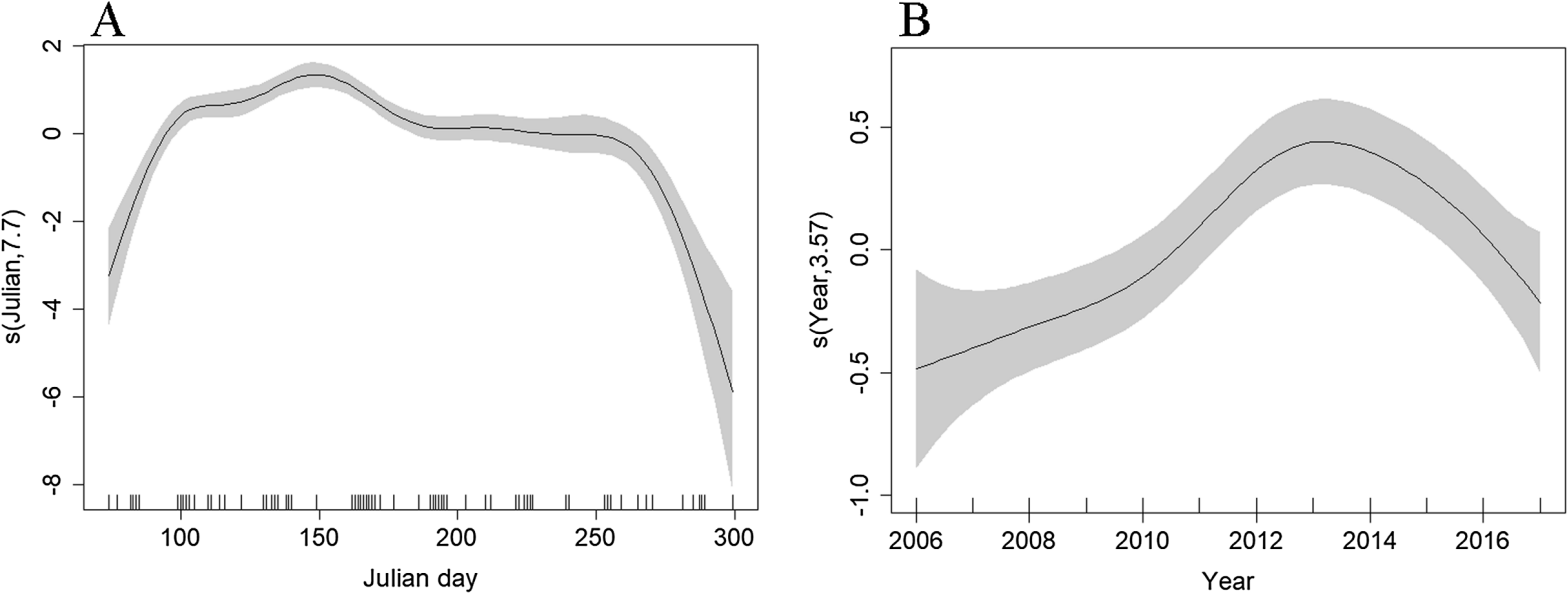
Plot of the GAM smooth fit of abundance across (A) Julian days and (B) Years in the Bay of Biscay. The solid line represents the best fit, with the gray shaded area representing the 95% confidence intervals. Vertical lines on the *x*-axis are the observed data values.

Depth (*p* < 0.001), distance to coast (*p* < 0.001), Julian day (*p* < 0.001), and year (*p* < 0.001) were all retained in the Tweedie DSM. The model explained a relatively low percentage of the deviance (13.3%) but passed model checks with a total CV of 0.072 (Fig. S4). There was an estimated density of 0.319 common dolphins per km^2^ (95% CI [0.277–0.367]). The highest densities were predicted to be towards the northern end of the surveyed region, close to the continental shelf edge with lower densities towards the Santander coast (Fig. 4). The effects of depth and distance to coast are less clear due to wide confidence intervals; however, density increased with increasing distance from the coast (Fig. S5), up to 2,000 m depth, then decreased at greater depths (Fig. S6). Similar to the Scilly’s route, numbers decreased towards winter (Fig. 7B). Densities appear to have increased between 2006 and 2013 and have decreased since (Fig. 7B).

## Discussion

### Common dolphin densities and trends

The highest densities of common dolphins were found in the small area surveyed in the Celtic Sea between Penzance and the Isles of Scilly, with an estimate of 0.40 per km^2^ (95% CI [0.305–0.524]). This is similar to the overall density estimated for the wider area of the Celtic Sea surveyed by the third SCANS survey (SCANS-III) in 2016 of 0.374 (95% CI [0.09–0.680]) (Hammond et al., 2017). However, it is important to note that the methods and areas covered by our study and that of SCANS differ, and broad trends should be compared throughout, rather than exact values. The mean group size is also similar between the two studies (9.68 in our study, and 10 in SCANS-III). The Bay of Biscay was estimated to have similarly high densities of common dolphins (0.319, with 95% CI [0.277–0.367]), which is considerably lower than that estimated by SCANS-III (0.784, with 95% CI [0.445–1.26]). This is likely to be due to the limited extent of the Bay of Biscay covered by the ferry route in comparison to the SCANS surveys which covered more of the off-shore waters and continental shelf edge—areas frequented by common dolphins and other cetacean species due to higher productivity along the shelf-edge (Hammond et al., 2009). Conversely, it is also possible that the density estimates reported in all regions of our study are over-estimates, as common dolphins are known to be attracted to vessels (Cañadas, Desportes & Borchers, 2004). Whilst animals were recorded when first seen, the occurrence of responsive movement could not be tested as these platforms of opportunity do not allow a double platform design. It is possible that some degree of the prevalence of animals recorded straight ahead was caused by responsive movement, as well as rounding of angles; and therefore some bias may arise here.

Common dolphins are infrequent visitors to the English Channel, as demonstrated by the low density estimated in this study (0.036 animals per km^2^ with 95% CI [0.024–0.05]), and absence of common dolphins recorded during the SCANS-III survey (Hammond et al., 2017). The role and importance of regular citizen science data collection is demonstrated particularly clearly here, allowing for the detection and monitoring of species in low-density areas which infrequent but extensive surveys may miss. This could be especially useful for endangered species, where low-densities may require important conservation action that could be critical to their continued presence. Platforms of opportunity facilitate regular monitoring that is unlikely to be practical with traditional means and can be used to survey data-deficient areas if infrastructure and logistics allow.

Densities of common dolphins on the Scilly’s route appear to have been relatively stable since 2006, until a decrease in 2017. This was the year with one of the highest number of stranded common dolphins on the Cornish coast in the past 15 years (Cornwall Wildlife Trust, 2018, personal communication). The decline in density in 2017 could be a result of the mass mortality of common dolphins before the start of the survey season or show a movement away from the survey area which may also be directly, or in-directly linked to the mass mortality event. But given the limited extent of the survey, it may just indicate a slight shift in distribution within the Celtic Sea, away from the sampled area, rather than a large scale change in distribution. If the decline continues, it may suggest that more research is needed to extend the data collection further into the Celtic Sea to explore these changes in density in more detail.

In the Bay of Biscay, higher densities were predicted in waters up to 2,500 m deep, with lower densities closer to the Santander coast, which is supported by previous studies (Kiszka et al., 2007; Hammond et al., 2009). Densities increased between 2006 and 2016, which is also supported by results from SCANS-II and SCANS-III (Hammond et al., 2013, 2017). However, our results suggest a decline from 2013 onwards which is similarly reported in Authier et al. (2018), which surveyed along the continental shelf. These decreasing or increasing trends as demonstrated by our data and supported by other studies, show the importance of long-term and frequent monitoring that can be provided by citizen science data, as infrequent surveys are not likely to identify finer-scale temporal changes in density and distribution.

Wide-scale infrequent surveys, such as the SCANS surveys (Hammond et al., 2001, 2013, 2017) can provide robust estimates of abundance which are essential for estimating the impacts of bycatch and other threats. These surveys also provide a complete snapshot of the distribution of the entire population at the time of survey (depending on the extent of the survey). However finer-scale spatial or temporal changes require additional monitoring. Without ongoing monitoring, which can be provided by citizen scientists or local dedicated projects, changes in distribution or abundance may remain unnoticed for an extended period. Ongoing monitoring has the potential to highlight changes and act as an early warning system, especially for a species such as common dolphins that are vulnerable to bycatch. Up-to-date information on distribution and trends is critical for appropriate and timely management of anthropogenic activities to ensure the conservation of vulnerable species.

### Benefits of citizen science data

Citizen science programs often have the potential to collect large quantities of data over a long period of time, and/or a wide area. The collection of long-term time series such as in this study is often not feasible for designed surveys which can be expensive, especially when chartering ships and paying running costs. Using platforms of opportunity such as ferries and cruise ships can make long-term surveys more affordable. Non-random survey designs, such as those imposed when surveying from ferries, limit inferences that can be made due to limited survey area; however, they are repeatedly sampled, providing extensive information on changes across that area over time. Temporal changes in density do need to be considered conservatively, especially in fixed areas covered by platforms of opportunity, as small-scale movements away from or into the survey area could influence these estimates considerably. However, these datasets can be important to inform wider-ranging survey design and form an early warning system about potential changes in the marine environment. Spatial and temporal trends identified by citizen science projects such as in this study can also be used by professional surveyors to determine suitable areas and times to survey their target species.

Conservation management requires up-to-date information to best conserve species. Many designed surveys are conducted infrequently, and citizen science data may allow regular evaluation of populations to inform policy makers and legislators. This is particularly relevant to species which don’t often warrant targeted surveys but face inter-annual variability of threats. One such example is the expected inter-annual changes in habitat use of common dolphins, and therefore variable overlap with fisheries that may lead to fluctuating bycatch rates. Whilst it is unlikely citizen science surveys will rival designed surveys for robust data collection, the two methodologies complement each other, with citizen science data filling in the gaps between designed surveys.

### Recommendations for high quality citizen science data

Citizen science can be a powerful monitoring tool; however, some datasets may possess certain challenges. To maximize the usability and power of citizen science datasets, simple measures can be taken. The following recommendations for high quality citizen science data are based on the authors’ experience working with citizen science data and are provided to hopefully improve the quality of similar data.

It is important to identify incomplete data or errors early in the data life-cycle. Early identification facilitates timely communication with the data collectors to correct the data where possible or provide further training to improve future data. To maintain quality, data should be checked for accuracy as it is collected in the field, with further exploration for broader patterns soon after the survey. If surveys are conducted as a team, an experienced individual should be responsible for checking that data are logical (e.g., angles are between 0° and 359°), and accurate (e.g., distances and angles are not rounded). In this study, angles were rounded to 0°; and subsequent training has addressed this practice to ensure future data are collected with as little bias as possible. A short cross-over period between recorders can be factored into the protocol, for example, when the survey team cycles through roles, the old recorder can discuss the current environmental conditions with the new observer to ensure consistency between recorders and continue training if required. When data are collected by lone citizen scientists without in situ discussion and checking of the data by others, further data validation rules may be required after collection. If the project allows, photographs of a subset of animals could be taken to confirm identification skills, or alternatively a digital quiz could be created to test survey skills and reinforce training.

Discussion should be nurtured, and the views of less experienced individuals should be welcomed. This allows their surveying techniques to be evaluated for accuracy; conversely inexperienced individuals are more likely to have recently undertaken structured training courses. If experienced recorders miss ongoing training, then there is a chance they could develop bad habits that vary from the intended protocol. It is important for citizen scientists to have a support network with ongoing training and avenues for queries to be addressed. Continued support could be in the form of face-to-face training days with active citizen scientists, mid-season reminders of successes and best practice, or annual training events.

In some cases, citizen science data can lack complete spatial coverage of the study area; however, there are often similar projects researching the same species. Coverage can be improved by combining similar datasets, for example the Joint Cetacean Protocol (Paxton et al., 2016) and the European Cetacean Monitoring Coalition (previously ARC; Brereton et al., 2001) collate data from many smaller-scale groups. Once data are converted into a shared format, an extensive dataset can be analyzed with greater spatial coverage. Collaborations such as these can be powerful and enhance monitoring to drive conservation of key species.

## Conclusions

We have demonstrated that citizen science data collected from platforms of opportunity have an important role to play in the continued monitoring of cetaceans. Many of the results are similar to those derived from wide-scale and robust, but infrequent surveys. Therefore, citizen science can complement traditional scientific monitoring by continuing monitoring between these surveys. Temporal changes in animal occurrence can be identified from regular surveys, which may allow dedicated surveys to further investigate the cause and degree of changes. If used appropriately, citizen science data can be used to identify changes in distribution or density which have conservation implications such as changing distributions that may cause an overlap with anthropogenic stressors.

## Supplemental Information

10.7717/peerj.8335/supp-1 Supplemental Information 1 Coefficient of variation of common dolphin density estimates in the English Channel.

10.7717/peerj.8335/supp-2 Supplemental Information 2 Coefficient of variation of common dolphin density estimates on the Scilly’s route.

10.7717/peerj.8335/supp-3 Supplemental Information 3 Plot of the GAM smooth fit of abundance with chlorophyll concentration on the Scilly’s route. The solid line represents the best fit, with the gray shaded area representing the 95% confidence intervals which widen with increasing concentrations. Vertical lines on the *x*-axis are the observed data values.

10.7717/peerj.8335/supp-4 Supplemental Information 4 Coefficient of variation of common dolphin density estimates in the Bay of Biscay.

10.7717/peerj.8335/supp-5 Supplemental Information 5 Plot of the GAM smooth fit of abundance with distance to coast in the Bay of Biscay. The solid line represents the best fit, with the gray shaded area representing the 95% confidence intervals. Vertical lines on the *x*-axis are the observed data values.

10.7717/peerj.8335/supp-6 Supplemental Information 6 Plot of the GAM smooth fit of abundance with depth in the Bay of Biscay. The solid line represents the best fit, with the gray shaded area representing the 95% confidence intervals, which are wide throughout. Vertical lines on the *x*-axis are the observed data values.

## Additional Information and Declarations

### Data Availability

The following information was supplied regarding data availability: The data are available on Mendeley Data: Robbins, James (2019), “Citizen science in the marine environment: A case-study estimating common dolphin densities in the north-east Atlantic”, Mendeley Data, v1 DOI 10.17632/xx4byv3d7k.1.

## References

[ref-1] Authier M, Dorémus G, Van Canneyt O, Boubert J-J, Gautier G, Doray M, Duhamel E, Massé J, Petitgas P, Ridoux V, Spitz J (2018). Exploring change in the relative abundance of marine megafauna in the Bay of Biscay, 2004–2016. Progress in Oceanography.

[ref-2] Becker EA, Forney KA, Ferguson MC, Foley DG, Smith RC, Barlow J, Redfern JV (2010). Comparing California current cetacean—habitat models developed using in situ and remotely sensed sea surface temperature data. Marine Ecology Progress Series.

[ref-3] Brereton T, Wall D, Cermeno P, Vasquez A, Curtis C, Williams A (2001). Cetacean monitoring in north-west European waters. http://www.marine-life.org.uk/media/27187/brereton_2001_arc%20report.pdf.

[ref-4] Buckland S, Anderson D, Burnham K, Laake J, Borchers D, Thomas L (2001). Introduction to distance sampling: estimating abundance of biological populations.

[ref-5] Buckland S, Rexsted E, Marques T, Oedekoven C (2015). Distance sampling methods and applications.

[ref-6] Cañadas A, Desportes G, Borchers D (2004). Estimation of g(0) and abundance of common dolphins (*Delphinus delphis*) from the NASS-95 Faroese survey. Journal of Cetacean Research and Management.

[ref-7] Cañadas A, Hammond PS (2008). Abundance and habitat preferences of the short-beaked common dolphin *Delphinus delphis* in the southwestern Mediterranean: implications for conservation. Endangered Species Research.

[ref-8] Cañadas A, Sagarminaga R, De Stephanis R, Urquiola E, Hammond PS (2005). Habitat preference modelling as a conservation tool: proposals for marine protected areas for cetaceans in southern Spanish waters. Aquatic Conservation: Marine and Freshwater Ecosystems.

[ref-9] Cañadas A, Sagarminaga R, García-Tiscar S (2002). Cetacean distribution related with depth and slope in the Mediterranean waters off southern Spain. Deep Sea Research Part 1: Oceanographic Research Papers.

[ref-10] Certain G, Ridoux V, van Canneyt O, Bretagnolle V (2008). Delphinid spatial distribution and abundance estimates over the shelf of the Bay of Biscay. ICES Journal of Marine Science.

[ref-11] Crall AW, Newman GJ, Stohlgren TJ, Holfelder KA, Graham J, Waller DM (2011). Assessing citizen science data quality: an invasive species case study. Conservation Letters.

[ref-12] Crosby A, Hawtrey-Collier A, Clear N, Williams R (2016). 2016 Annual summary report: marine strandings in Cornwall and the Isles of Scilly. http://www.cornwallwildlifetrust.org.uk/sites/default/files/2016_summary_report_final_-_marine_strandings_in_cornwall_and_the_isles_of_scilly.pdf.

[ref-13] Davies TK, Stevens G, Meekan MG, Struve J, Rowcliffe JM (2013). Can citizen science monitor whale-shark aggregations? Investigating bias in mark-recapture modelling using identification photographs sourced from the public. Wildlife Research.

[ref-14] De Boer M, Leaper R, Keith S, Simmonds M (2008). Winter abundance estimates for the common dolphin (*Delphinus delphis*) in the western approaches of the English Channel and the effect of responsive movement. Journal of Marine Animals and Their Ecology.

[ref-15] Embling CB, Walters AEM, Dolman SJ (2015). How much effort is enough? The power of citizen science to monitor trends in coastal cetacean species. Global Ecology and Conservation.

[ref-16] EMODnet Bathymetry Consortium (2016). EMODnet Digital Bathymetry (DTM 2016).

[ref-17] ESRI (2017). ArcGIS Desktop: Release 10.5.

[ref-18] Gordon J (2001). Measuring the range to animals at sea from boats using photographic and video images. Journal of Applied Ecology.

[ref-19] Hammond PS, Berggren P, Benke H, Borchers DL, Collet A, Heide-Jørgensen MP, Heimlich S, Hiby AR, Leopold MF, Øien N (2001). Abundance of harbour porpoise and other cetaceans in the North Sea and adjacent waters. Journal of Applied Ecology.

[ref-20] Hammond P, Lacey C, Gilles A, Viquerat S, Börjesson P, Herr H, Macleod K, Ridoux V, Santos M, Scheidat M, Teilmann J, Vingada J, Øien N (2017). Estimates of cetacean abundance in European Atlantic waters in summer 2016 from the SCANS-III aerial and shipboard surveys. https://synergy.st-andrews.ac.uk/scans3/files/2017/05/SCANS-III-design-based-estimates-2017-05-12-final-revised.pdf.

[ref-21] Hammond PS, Macleod K, Berggren P, Borchers DL, Burt L, Cañadas A, Desportes G, Donovan GP, Gilles A, Gillespie D, Gordon J, Hiby L, Kuklik I, Leaper R, Lehnert K, Leopold M, Lovell P, Øien N, Paxton CGM, Ridoux V, Rogan E, Samarra F, Scheidat M, Sequeira M, Siebert U, Skov H, Swift R, Tasker ML, Teilmann J, Van Canneyt O, Vázquez JA (2013). Cetacean abundance and distribution in European Atlantic shelf waters to inform conservation and management. Biological Conservation.

[ref-22] Hammond P, MacLeod K, Gillespsie D, Swift R, Winship A, Burt M, Cañadas A, Vazquez J, Ridoux V, Certain G, Van Canneyt O, Lens S, Santos B, Rogan E, Uriarte A, Hernandez C, Castro R (2009). Cetacean offshore distribution and abundance in the European Atlantic (CODA). http://biology.st-andrews.ac.uk/coda/documents/CODA_Final_Report_11-2-09.pdf.

[ref-23] Hijmans R (2017). https://CRAN.R-project.org/package=raster.

[ref-24] Hyder K, Townhill B, Anderson LG, Delany J, Pinnegar JK (2015). Can citizen science contribute to the evidence-base that underpins marine policy?. Marine Policy.

[ref-25] Kiszka J, Macleod K, Van Canneyt O, Walker D, Ridoux V (2007). Distribution, encounter rates, and habitat characteristics of toothed cetaceans in the Bay of Biscay and adjacent waters from platform-of-opportunity data. ICES Journal of Marine Science.

[ref-26] MacLeod CD, Brereton T, Martin C (2009). Changes in the occurrence of common dolphins, striped dolphins and harbour porpoises in the English Channel and Bay of Biscay. Journal of the Marine Biological Association of the United Kingdom.

[ref-27] Mate BR, Rossbach KA, Nieukirk SL, Wells RS, Blair Irvine A, Scott MD, Read AJ (1995). Satellite-monitored movemens and dive behaviour of a bottlenose dolphin (*Tursiops truncatus*) in Tampa Bay, Florida. Marine Mammal Science.

[ref-28] McClellan CM, Brereton T, Dell’Amico F, Johns DG, Cucknell A-C, Patrick SC, Penrose R, Ridoux V, Solandt J-L, Stephan E, Votier SC, Williams R, Godley BJ, Bograd SJ (2014). Understanding the distribution of marine megafauna in the English Channel region: identifying key habitats for conservation within the busiest seaway on earth. PLOS ONE.

[ref-29] Miller D (2017). https://CRAN.R-project.org/package=Distance.

[ref-30] Miller DL, Burt ML, Rexstad EA, Thomas L, Gimenez O (2013). Spatial models for distance sampling data: recent developments and future directions. Methods in Ecology and Evolution.

[ref-31] Miller D, Rexstad R, Burt L, Bravington M, Hedley S (2017). https://CRAN.R-project.org/package=dsm.

[ref-32] Miller D, Rexstad E, Thomas L, Marshall L, Laake J (2018). Distance sampling in R.

[ref-33] Moura A, Sillero N, Rodrigues A (2012). Common dolphin (*Delphinus delphis*) habitat preferences using data from two platforms of opportunity. Acta Oecologica.

[ref-34] NASA, Ocean Biology Ecology Laboratory (2017). Moderate-resolution Imaging Spectroradiometer (MODIS) Aqua. https://oceancolor.gsfc.nasa.gov/data/aqua/.

[ref-35] Paxton C, Scott-Hayward L, Mackenzie M, Rexstad E, Thomas L (2016). Revised phase III data analysis of Joint Cetacean Protocol data resource. http://jncc.defra.gov.uk/pdf/JNCC_Report_517_FINAL_web.pdf.

[ref-36] Peltier H, Authier M, Deaville R, Dabin W, Jepson PD, van Canneyt O, Daniel P, Ridoux V (2016). Small cetacean bycatch as estimated from stranding schemes: the common dolphin case in the northeast Atlantic. Environmental Science & Policy.

[ref-37] Peltier H, van Canneyt O, Dabin W, Dars C, Demaret F, Ridoux V (2017). New fishery-related unusual mortality and stranding events of common dolphins in the Bay of Biscay, February–March 2017, France. http://uk.whales.org/sites/default/files/attachment/news/2017/06/peltier_2017_french_bycatch_related_mass_mortality_sc_67a_him_wp_08.pdf.

[ref-38] Pierce D (2017). https://CRAN.R-project.org/package=ncdf4.

[ref-39] Postles M, Bartlett M (2018). The rise of BioBlitz: evaluating a popular event format for public engagement and wildlife recording in the United Kingdom. Applied Environmental Education & Communication.

[ref-40] R Development Core Team (2017). R: a language and environment for statistical computing.

[ref-41] Roberts JJ, Best BD, Dunn DC, Treml EA, Halpin PN (2010). Marine geospatial ecology tools: an integrated framework for ecological geoprocessing with ArcGIS, Python, R, MATLAB, and C++. Environmental Modelling & Software.

[ref-42] Rogan E, Mackey M (2007). Megafauna bycatch in drift nets for albacore tuna (*Thunnus alalonga*) in the NE Atlantic. Fisheries Research.

[ref-43] Spitz J, Chouvelon T, Cardinaud M, Kostecki C, Lorance P (2013). Prey preferences of adult sea bass *Dicentrarchus labrax* in the northeastern Atlantic: implications for bycatch of common dolphin *Delphinus delphis*. ICES Journal of Marine Science.

[ref-44] Sullivan BL, Wood CL, Iliff MJ, Bonney RE, Fink D, Kelling S (2009). eBird: a citizen-based bird observation network in the biological sciences. Biological Conservation.

[ref-45] Thomas L, Buckland ST, Rexstad EA, Laake JL, Strindberg S, Hedley SL, Bishop JRB, Marques TA, Burnham KP (2010). Distance software: design and analysis of distance sampling surveys for estimating population size. Journal of Applied Ecology.

[ref-46] Tonachella N, Nastasi A, Kaufman G, Maldini D, Rankin RW (2012). Predicting trends in humpback whale (*Megaptera novaeangliae*) abundance using citizen science. Pacific Conservation Biology.

[ref-47] Vermeiren P, Munoz C, Zimmer M, Sheaves M (2016). Hierarchical toolbox: ensuring scientific accuracy of citizen science for tropical coastal ecosystems. Ecological Indicators.

[ref-48] Williams R, Hedley SL, Hammond PS (2006). Modelling distribution and abundance of Antarctic baleen whales using ships of opportunity. Ecology and Society.

[ref-49] Wood S (2006). Generalized additive models (Texts in statistical science).

