# Supplementary figures and images for: Citizen science in the marine environment: estimating common dolphin densities in the north-east Atlantic
Source: PeerJ. 2020 Feb 28;8:e8335. doi: 10.7717/peerj.8335 (PMC7050547; doi:10.7717/peerj.8335)

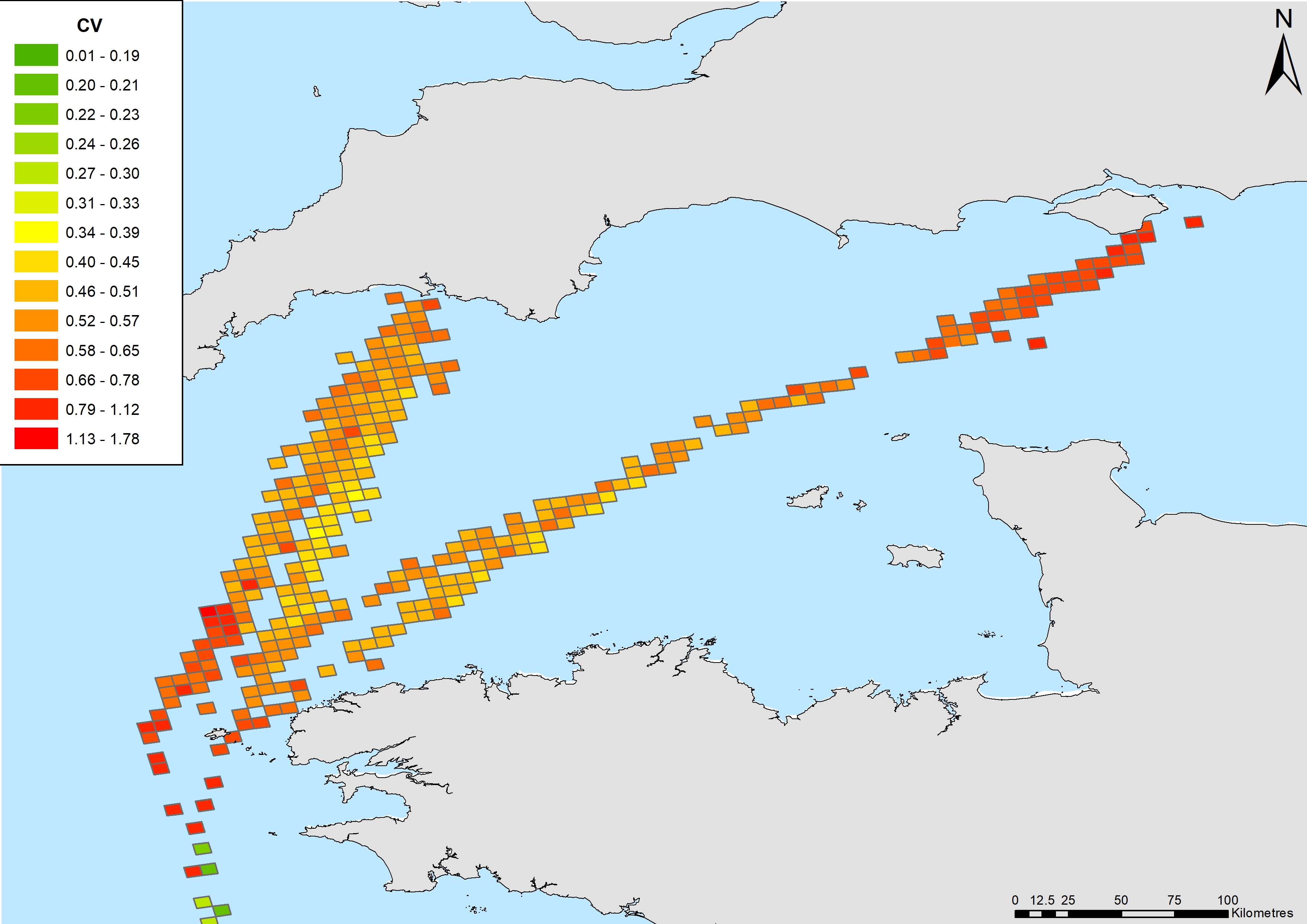

Supplement: Supplemental Information 1 [file peerj-08-8335-s001.jpg]

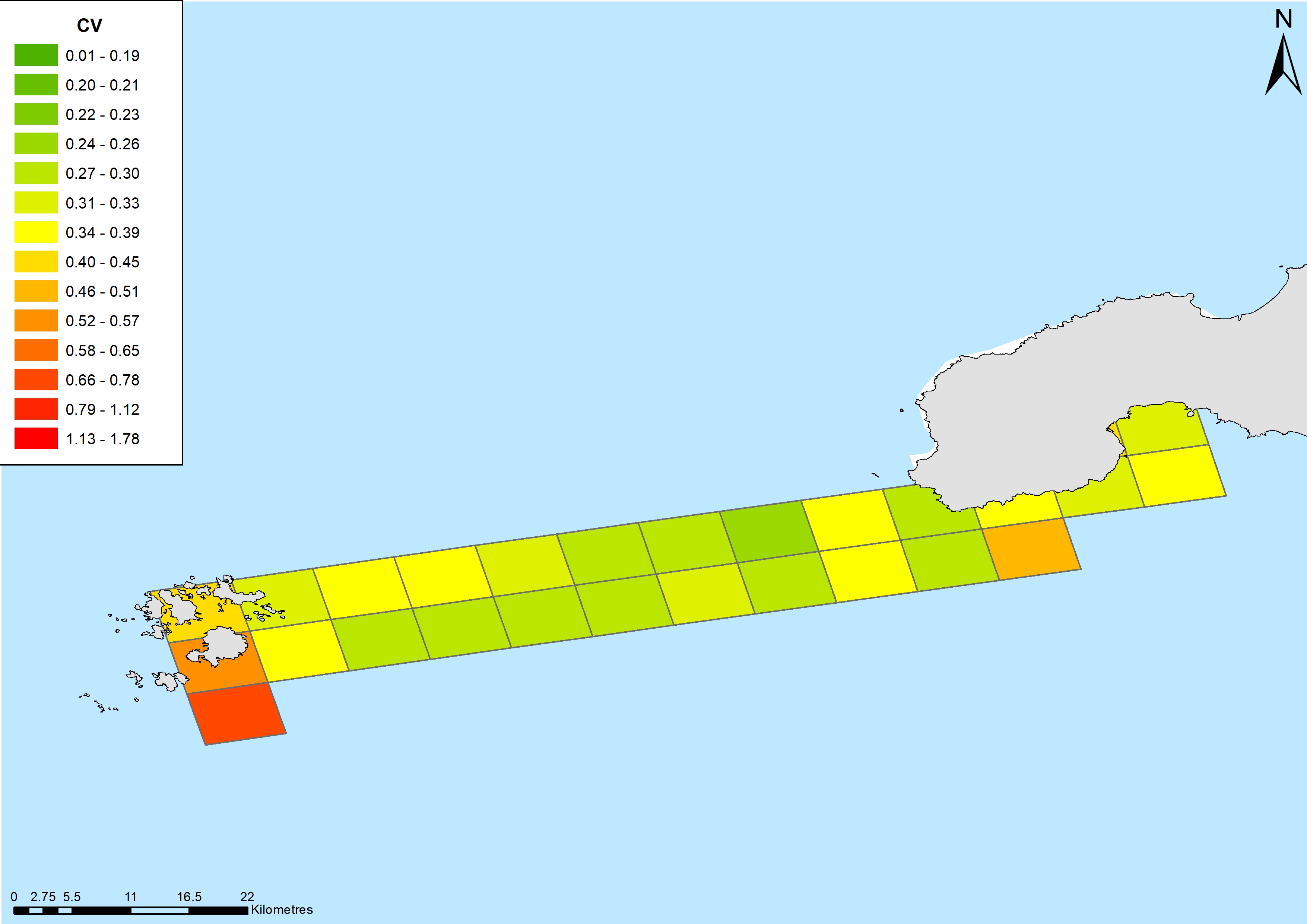

Supplement: Supplemental Information 2 [file peerj-08-8335-s002.jpg]

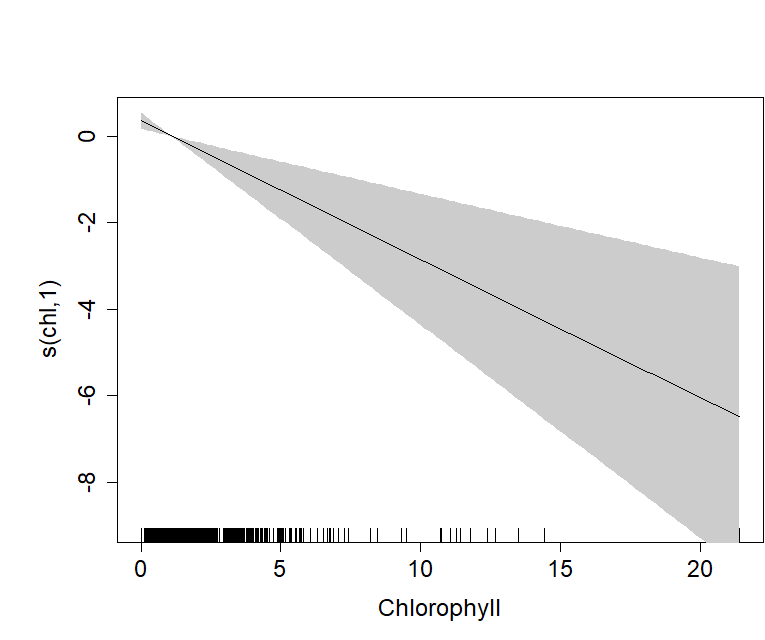

Supplement: Supplemental Information 3 — The solid line represents the best fit, with the gray shaded area representing the 95% confidence intervals which widen with increasing concentrations. Vertical lines on the x-axis are the observed data values. [file peerj-08-8335-s003.png]

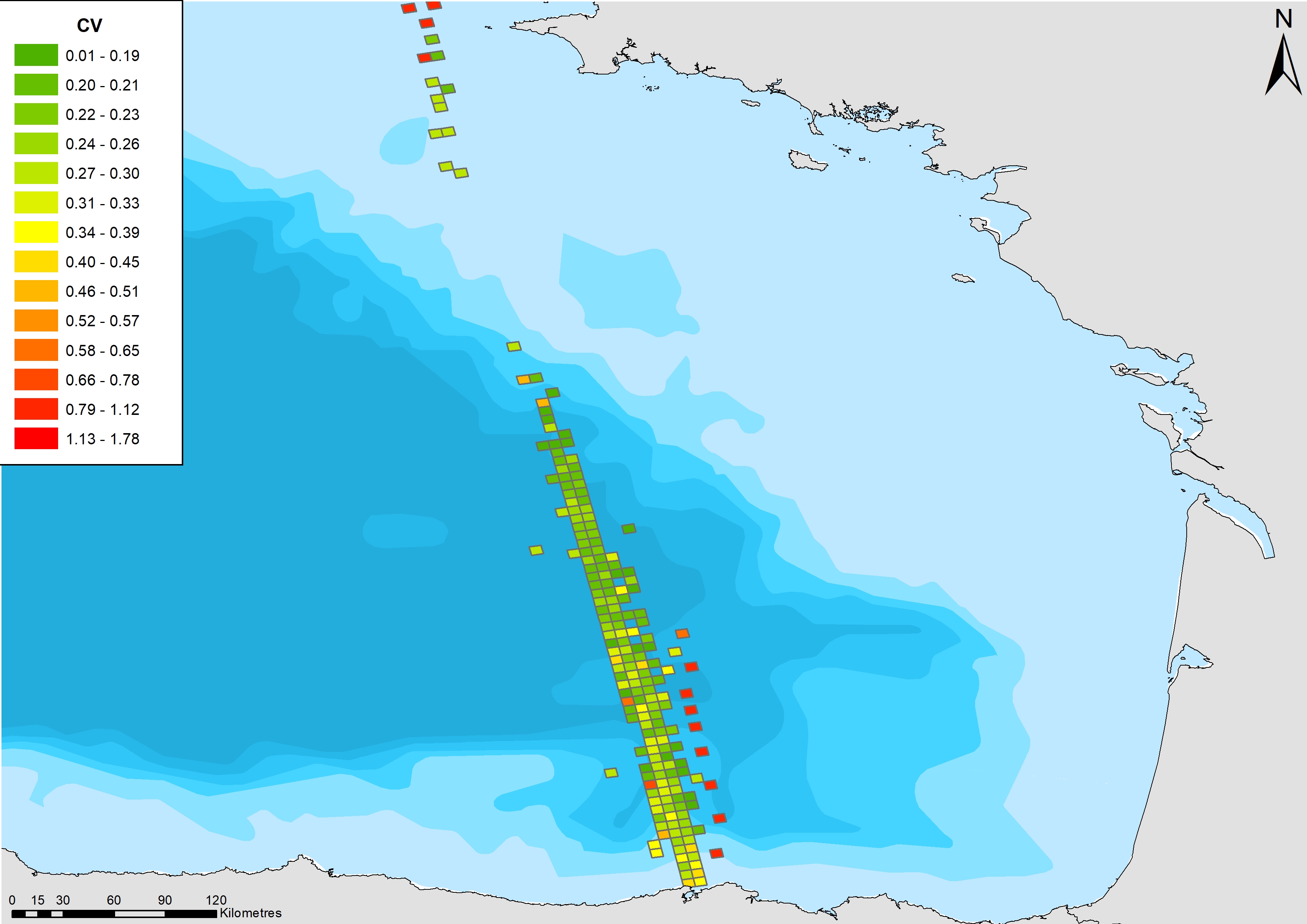

Supplement: Supplemental Information 4 [file peerj-08-8335-s004.jpg]

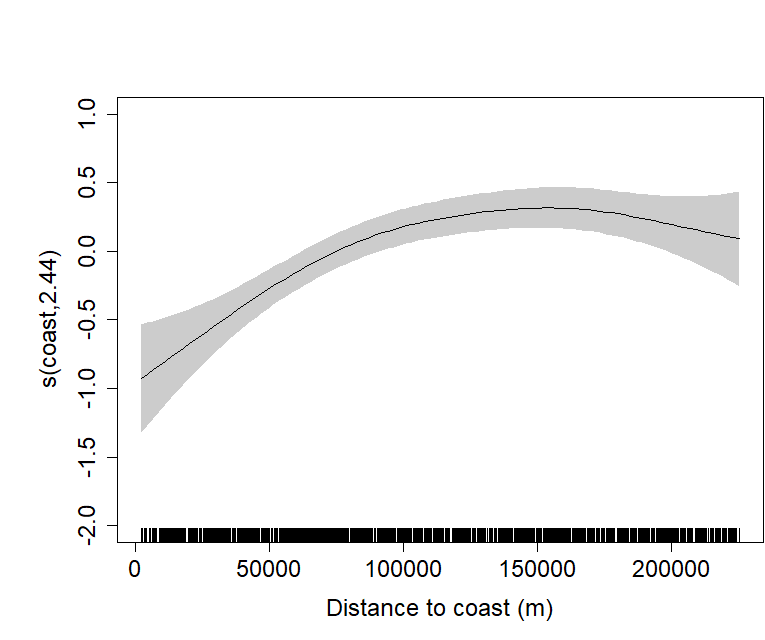

Supplement: Supplemental Information 5 — The solid line represents the best fit, with the gray shaded area representing the 95% confidence intervals. Vertical lines on the x-axis are the observed data values. [file peerj-08-8335-s005.png]

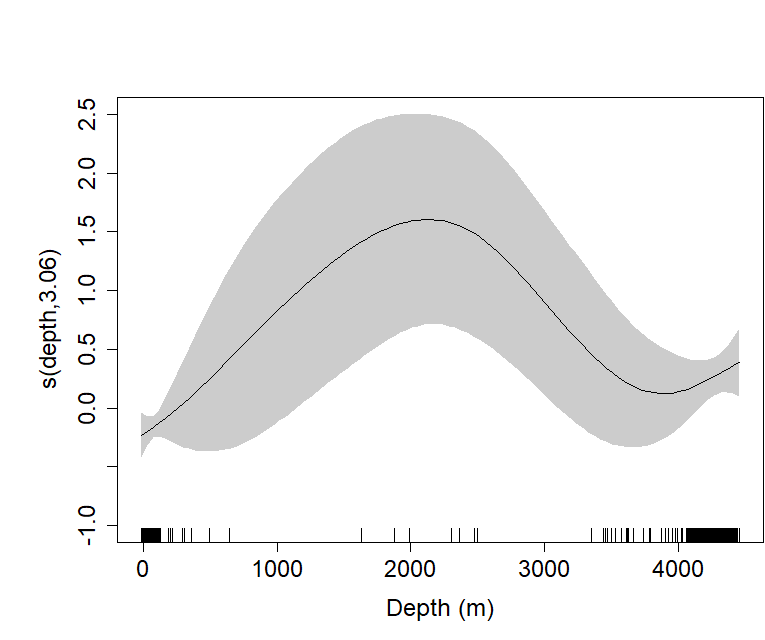

Supplement: Supplemental Information 6 — The solid line represents the best fit, with the gray shaded area representing the 95% confidence intervals, which are wide throughout. Vertical lines on the x-axis are the observed data values. [file peerj-08-8335-s006.png]
